# Oscillatory rhythm of reward: anticipation and processing of rewards in children with and without autism

**DOI:** 10.1186/s13229-018-0189-5

**Published:** 2018-01-30

**Authors:** Katherine Kuhl-Meltzoff Stavropoulos, Leslie J. Carver

**Affiliations:** 1 Riverside Graduate School of Education, University of California, 9500 University Avenue, Riverside, CA 92521 USA; 2 University of California, San Diego, USA

**Keywords:** Autism spectrum disorder, Alpha asymmetry, Theta, Reward processing, Social stimuli

## Abstract

**Background:**

Autism spectrum disorder (ASD) is a complex neurodevelopmental condition, and multiple theories have emerged concerning core social deficits. While the social motivation hypothesis proposes that deficits in the social reward system cause individuals with ASD to engage less in social interaction, the overly intense world hypothesis (sensory over-responsivity) proposes that individuals with ASD find stimuli to be too intense and may have hypersensitivity to social interaction, leading them to avoid these interactions.

**Methods:**

EEG was recorded during reward anticipation and reward processing. Reward anticipation was measured using alpha asymmetry, and post-feedback theta was utilized to measure reward processing. Additionally, we calculated post-feedback alpha suppression to measure attention and salience. Participants were 6- to 8-year-olds with (*N* = 20) and without (*N* = 23) ASD.

**Results:**

Children with ASD showed more left-dominant alpha suppression when anticipating rewards accompanied by nonsocial stimuli compared to social stimuli. During reward processing, children with ASD had less theta activity than typically developing (TD) children. Alpha activity after feedback showed the opposite pattern: children with ASD had greater alpha suppression than TD children. Significant correlations were observed between behavioral measures of autism severity and EEG activity in both the reward anticipation and reward processing time periods.

**Conclusions:**

The findings provide evidence that children with ASD have greater approach motivation prior to nonsocial (compared to social) stimuli. Results after feedback suggest that children with ASD evidence less robust activity thought to reflect evaluation and processing of rewards (e.g., theta) compared to TD children. However, children with ASD evidence greater alpha suppression after feedback compared to TD children. We hypothesize that post-feedback alpha suppression reflects general cognitive engagement—which suggests that children with ASD may experience feedback as overly intense. Taken together, these results suggest that aspects of both the social motivation hypothesis and the overly intense world hypothesis may be occurring simultaneously.

## Background

Autism spectrum disorders (ASD) are characterized by impairments in two broad categories: social communication (including both verbal and non-verbal communication), and presence of restricted interests and/or repetitive behaviors [1]. Given that autism is hypothesized to be neurologically based [2, 3], it is not surprising that theories have attempted to identify underlying neural systems that account for this complex condition. In order to accurately identify neural systems that might be of interest, researchers turn to hypotheses concerning the underlying causes of symptoms of ASD. Although many theories of ASD have been proposed, of particular relevance to the current investigation are two alternative theories: the social motivation hypothesis [4–6] and sensory over-responsivity [7–9] as described by the overly intense world hypothesis [10, 11].

The social motivation hypothesis (SMH) proposes that individuals with ASD are not driven to seek out or engage in social interaction because those interactions are not as rewarding for them as they are to their typically developing (TD) peers. The hypothesis states that less social interaction during critical periods of development leads to abnormal neural specialization, which can affect cognitive development and lead to fewer social interactions over time.

Given that a central assumption of the SMH is that social interactions are not as rewarding for children with ASD as they are for TD children, previous investigations of the hypothesis have measured neural responses to social versus nonsocial stimuli in children and adolescents with and without ASD. Whereas the SMH supposes *hypo*activation of the reward system for social stimuli, the intense world hypothesis (IWH) posits that individuals with ASD experience neural *hyper*reactivity, which leads to the inability to “gate” information flow and selectively attend to information. Overall, the IWH argues that individuals with ASD perceive the world as presenting overwhelming multisensory stimulation. With regard to social deficits, the IWH notes that because social situations are particularly complex and difficult to predict, individuals with ASD find them particularly intense and unpleasant, which leads to withdrawal or self-soothing behaviors [10]. Thus, while the SMH implicates the reward system as a critical neural mechanism underlying social deficits in ASD, the IWH implicates sensory and/or attentional systems underlying behavioral patterns in ASD.

Although the SMH and IWH appear quite different (and potentially contradictory insofar as they hypothesize different neural mechanisms in ASD), the current investigation attempts to explore whether these theories could exist in tandem. In this view, both reduced social rewards and overwhelming responses to social stimuli could co-exist. The approach we take utilizes a reward-related paradigm that allows us to separate the effects of reward *anticipation* from reward *processing* [12].

Specifically, we hypothesize that the SMH will hold true for periods of reward anticipation—when individuals with ASD are waiting for a social reward. According to the SMH account, individuals with ASD will evidence less anticipatory reward-related brain activity compared to TD individuals when anticipating social rewards. We hypothesize *hypoactivation* of social reward anticipation as this appears concordant with behavioral observations of ASD symptoms. That is, individuals with ASD are less likely to initiate social engagement with others, which we hypothesize may be due to aberrant reward anticipation for social information. In contrast to typically developing individuals, people with ASD may not expect social interactions to be inherently rewarding and therefore may be less likely to initiate such interactions. However, we simultaneously hypothesize that after rewards are delivered (e.g., during reward *processing*), individuals with ASD will show signs of neural *hyperreactivity*, providing evidence for the IWH. In line with the IWH, we hypothesize that individuals with ASD may be overwhelmed by the social stimuli provided in the feedback phase. If social stimuli are aversive to individuals with ASD, then in addition to evidencing reduced reward-related anticipation, individuals with ASD may overreact to them when they are presented.

Previous neuroscience research on reward anticipation and processing in ASD has utilized both electrophysiology and functional magnetic resonance (fMRI) imaging. As the current manuscript focuses on electrophysiology, we will not review the fMRI literature in detail. Note, however, that there have been a number of manuscripts exploring social and nonsocial reward anticipation and processing in ASD using fMRI. Findings of these studies are mixed, with some finding evidence of global deficits in reward responsiveness for individuals with ASD [13–15], and others suggesting that responses to social rewards are diminished [16–18].

Our previous research has used event-related potentials (ERP), which measure time-locked neural activity averaged over multiple trials. However, the published reward-related literature demonstrates that interesting information can also be gained from exploring event-related spectral perturbations (ERSP). ERSP measures can provide information about brain activity patterns in single trials rather than averaging activity over multiple trials, which is necessary to observe patterns of activity that are not both time and phase-locked. In this way, ERSP measures can provide information beyond what can be observed using more traditional ERP measures. ERSP measures stimulus-related modulation of power in the EEG signal relative to baseline. Differences in EEG power are of interest in ASD, as this oscillatory electrical activity is hypothesized to involve inhibitory processes and activity of GABAergic interneurons [19]. Disruption of inhibitory activity has been proposed as an explanation for symptoms commonly observed in ASD (for a review, see [20]).

### Reward anticipation

Previous research suggests that anticipation of feedback is related to the suppression of activity in the alpha band (8–12 Hz). Studies of both visual and auditory modalities have found alpha power suppression prior to feedback on a time-estimation task [21, 22]. However, of particular relevance to motivation and reward anticipation is alpha band asymmetry. Decades of research have focused on asymmetry in EEG activity between the right and left hemispheres (particularly increased left versus right hemisphere activity) to indicate reward sensitivity and approach motivation [23–26]. Over two decades ago, researchers found evidence that left-dominant alpha suppression occurred more robustly during anticipation of reward versus punishment trials [23]. Conversely, right-dominant alpha suppression was observed during anticipation of punishment relative to reward trials. The authors hypothesized that left-dominant alpha suppression was an accurate marker for approach motivation in healthy adults.

#### Reward anticipation and autism spectrum disorder

Although much of the previous research concerning alpha asymmetry in individuals with psychiatric diagnoses has focused on depression and schizophrenia, recent attention has been given to the reward system in autism spectrum disorder (ASD). Of the studies that have directly measured reward anticipation in ASD, none have measured stimulus-locked alpha asymmetry. Rather, research has used event-related-potential (ERP) measures of reward anticipation. Two ERP components have been studied: the stimulus preceding negativity (SPN) and P300. The SPN is a negative slow-wave component thought to reflect reward expectation and activity in the dopaminergic reward system [27]. The P300 is thought to index attentional orienting and stimulus salience [28, 29].

Of the previous studies measuring reward anticipation in ASD, one found that both children with ASD and attention-deficit hyperactivity disorder (ADHD) evidenced a larger stimulus preceding negativity (SPN) component compared to their TD peers when anticipating positive outcomes, but equivalent activity when anticipating negative outcomes [30]. A second group found that TD children had greater P300 activity when anticipating reward versus nonreward conditions, whereas children with ASD did not [31]. Our own previous results measured the SPN component during anticipation of social versus nonsocial rewards and found that children with ASD evidenced a smaller SPN when anticipating social rewards compared to their TD peers [12].

Studies of reward anticipation in ASD have not utilized stimulus-locked measures of oscillatory activity (e.g., event-related spectral perturbations, ERSP). To our knowledge, no studies have been conducted on alpha asymmetry in ASD during social reward anticipation. We note, however, that there have been previous EEG studies of resting asymmetry in ASD (e.g., brain activity measured “at rest,” while the subject is not watching or listening to specific stimuli). For example, [32] explored the relationship between resting frontal asymmetry and social symptoms of ASD. The authors found that children with ASD with left dominant frontal asymmetry displayed less severe social impairments compared to children with right frontal asymmetry. The authors interpreted this finding as consistent with the hypothesis that left asymmetry is related to approach motivation whereas right asymmetry appears related to withdrawal. More recently, [33] also studied resting EEG asymmetry in ASD. The authors found children with ASD with left asymmetry had less severe social deficits, but this effect was mediated by verbal IQ. The authors also found that parents of children who demonstrated left dominant asymmetry reported later age of symptom onset compared to the age of onset reported by parents whose children had right dominant asymmetry.

### Reward processing

Whereas the suppression and asymmetry of alpha-band activity is thought to reflect anticipation of rewards, another important consideration in research related to reward is reward processing. Reward processing occurs after feedback and has been measured using both EEG and event-related potentials (ERP). Previous ERP research suggests that the feedback-related negativity (FRN) relates to reward processing and may reflect processes related to expected versus actual rewards [34]. Less research has been done on neural oscillations related to reward processing, but extant studies point to enhancement of theta band (4–8 Hz) activity as a likely candidate to reflect reward processing. Post-stimulus theta appears sensitive to reward evaluation [35], and previous studies have measured both the FRN and theta as they are hypothesized to reflect similar neural processes [36]. Finally, as has been observed in the FRN component, theta appears to be stronger for negative feedback compared to positive feedback [37, 38] and is stronger when feedback reflects a higher magnitude of reward [39].

#### Reward processing and autism spectrum disorder

Studies of reward processing in ASD are more plentiful than those of reward anticipation, although few studies have explored reward processing in ASD using *social* stimuli. Two previous ERP studies comparing the feedback-related negativity (FRN) component of individuals with and without ASD suggest that individuals with ASD do not demonstrate significant differences in feedback processing for nonsocial rewards [40, 41]. However, most studies of the FRN utilize nonsocial reward paradigms (i.e., paradigms with monetary rewards). In our previous work comparing social versus nonsocial rewards in ASD, we found differences in how children with and without ASD respond to feedback indicating correct or incorrect performance on a guessing task, compared to their TD peers [12]. Importantly, no studies to our knowledge have measured ERSPs during social reward processing in children with and without ASD.

Although not directly related to reward processing, there is a body of literature on oscillatory activity and ASD in response to social stimuli (e.g., after stimuli have been presented). Although these tasks were not designed to elicit activity of the reward system, they are relevant to the current investigation as they provide information about oscillatory activity in ASD in response to faces and will be reviewed briefly. Dawson and colleagues [42] measured both alpha and theta-band activity after a 2-year behavioral intervention (early start Denver model; ESDM) designed to improve social skills of toddlers with ASD. The authors interpreted oscillatory activity in these two bands as a marker of general cognitive engagement and cortical activity, arguing that greater alpha suppression and enhanced theta-band activity suggest enhanced cortical activation. Findings suggested that toddlers with ASD who participated in ESDM “normalized” their degree of theta and alpha band EEG activity in response to repeated images of faces. In a study of adults with Asperger syndrome (AS), researchers observed lower delta/theta synchronization in temporal and occipital-parietal regions in the AS versus control groups in response to emotional faces [43]. The authors interpreted these differences to reflect difficulty of individuals with AS with implicit emotional face recognition, as previous literature suggests both delta and theta are involved with limbic-cortical connections. This is particularly interesting due to findings suggesting delta/theta synchronization is associated with nonconscious versus conscious face recognition [44]. Therefore, the authors conclude that this pattern of oscillatory activity underscores difficulties individuals with AS experience when identifying emotional faces (e.g., individuals with AS must rely on cognitive, rather than implicit, processes to correctly identify facial expressions). In a different study of adults with AS, results suggested less theta activity, but increased activity in the beta2 range (16–20 Hz) after viewing faces compared to a control group [45]. The authors interpreted enhanced beta2 in the AS to reflect greater reliance on voluntary attention and cognitive processes during facial recognition compared to controls, and decreased theta activity to reflect abnormalities in thalamic-cortical and hippocampal-cortical circuits, as well as potential abnormalities in amygdala activity in response to faces in AS. These studies underscore the utility of measuring oscillatory activity in individuals with ASD and provide information about potential differences between typically developing individuals and those with ASD in response to emotional faces.

### Current study

The current study was conducted to gain understanding of event-related spectral perturbations (ERSP) in children with and without ASD for social reward anticipation and reward processing. We are unaware of any previous investigations that have measured ERSP in this population during both reward anticipation and reward processing. In addition, the current study will add to the literature comparing brain activity of children with and without ASD in response to social versus nonsocial rewards.

Consistent with the SMH, we hypothesized that children with ASD would evidence less left-dominant alpha suppression when anticipating social rewards compared to their TD peers, as this would reflect less approach motivation. Similarly, we expected that children with ASD would evidence less theta-band activity in response to social rewards compared to their TD peers, as activity in the theta band after feedback is thought to reflect reward processing. However, we also hypothesized that we would observe enhanced alpha-band suppression in children with ASD during reward processing and argue that this would provide evidence in favor of the IWH as alpha band suppression after stimulus presentation is thought to reflect cortical activity and cognitive engagement [42]. We postulate that if theta-band activity was *hypo*active in ASD during reward processing, but alpha band suppression was *hyper*active, it would provide initial evidence that reward-related activity in ASD is under-active while attentional processes are over-active. Finally, we hypothesized that measures of social behavior would be correlated with alpha asymmetry (during reward anticipation), and both theta and alpha activity (during reward processing).

We previously reported the results of event-related potential (ERP) brain activity from the cohort of children with ASD in the current investigation [12]. The current manuscript reports the results of a novel analysis designed to address the specific predictions of the SMH and the IWH theories regarding the reward system in ASD.

## Methods

### Stimuli and task

The stimuli and task are described in detail in [12, 46]. Briefly, the task was a guessing game that presented blocks of trials that used left and right visual stimuli (question marks). Participants were asked to indicate their guess via button press whether the left or right stimulus was “correct.” After this choice, the left and right question marks were replaced with an arrow in the middle pointing towards whichever question mark the participant chose. This was done to reinforce the idea that participants had control over the task and their responses were being recorded.

There were two blocked feedback conditions: social versus nonsocial. Incidental stimuli in the social condition were faces obtained from the NimStim database [47] that were smiling for “correct” answers and frowning for “incorrect” answers. To avoid confounds resulting from use of a single face or gender, 33 faces (18 female, 15 male) from the database were utilized. Incidental stimuli in the nonsocial condition were composed of scrambled face elements from the social condition formed into an arrow that pointed upwards for “correct” answers and downwards for “incorrect” answers. The use of scrambled faces to construct the arrow controlled for low-level visual features of the stimuli. Images were scrambled using the Adobe Photoshop “scramble” filter. This filter breaks images into square blocks and rearranges them randomly. The scrambled face images were then made into the shape of an arrow using Photoshop.

Both faces and arrows were presented in pseudorandom order, with no image repeating on consecutive trials (e.g., participants never saw the same face or arrow as “correct” or “incorrect” more than once in a row). Presented stimuli had a horizontal visual angle of 14.5° and a vertical visual angle of 10.67°. Each participant viewed identical stimuli in the same order for each condition (e.g., the social feedback block was the same for each participant), but whether individuals viewed the social versus nonsocial block first was counterbalanced between participants.

**Fig. 1:**
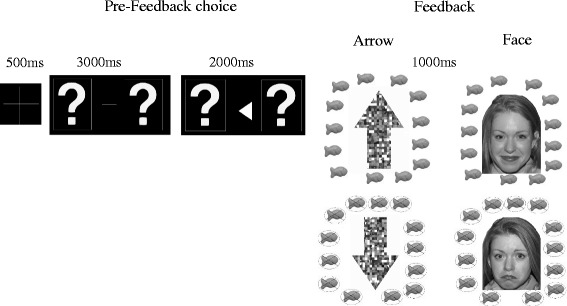
Stimulus presentation and timing. Feedback for the social condition is shown in the left column, and feedback for the nonsocial condition is shown in the right column. Feedback for “correct” answers is shown on top, and feedback for “incorrect” answers is shown below

Participants were told that the reward for each correct answer was a goldfish cracker, or if they preferred, fruit snacks. Participants were told there was no penalty for incorrect answers. Participants were told that if they guessed correctly, they would see a ring of intact goldfish crackers, and the goldfish would be crossed out for incorrect answers. Importantly, in both the social and nonsocial feedback trials, the face/arrow information was incidental. Figure 1 depicts the stimuli and timeline in the social and nonsocial conditions. A computer program predetermined correct versus incorrect answers in pseudorandom order such that children got 50% “correct” and 50% “incorrect,” with no more than three of the same answer in a row.

The two feedback conditions (face/“social” trials and arrow/“nonsocial” trials) were tested in separate blocks, each composed of 80 trials. Within each block of 80 trials, there were 30-s breaks every 15 trials. During breaks, participants were asked to relax or move if they felt restless. Between blocks, a longer break (5–10 min) was taken. To control for attentional effects, children were observed via webcam, and trials in which they were not attending to the stimulus were marked and discarded during analysis. Of the final sample, three children had trials excluded for this reason, and of those three, none had more than 10 trials excluded in this way.

### Participants

We tested TD children (*N* = 23) and children with ASD (*N* = 20). Exclusionary criteria for participants with ASD included history of seizures, brain injury, neurological disorders, or any concurrent psychiatric condition (other than ASD), based on parent report. Exclusionary criteria for TD participants included all of the above criteria, plus an immediate family history of ASD. None of the children in the TD group were taking psychoactive medications. Three children in the ASD group were taking medication in order to improve concentration, but one of the three did not take his medication on the day he came in for the current study. Participants were recruited from a UC San Diego subject pool and through postings on websites for parents of children on the autism spectrum. All participants had normal hearing and normal or corrected to normal vision. Procedures were approved by the institutional review board, and written consent was obtained from caregivers. All children over 7 years of age signed an assent form.

IQ scores [48] were available for all 20 children with ASD, and 22 of 23 TD children (one TD child was unable to complete the WASI due to time constraints). Of the final sample of 43 children, no significant differences were found between groups on full scale IQ scores, *F*(1,40) = .36. There were differences between the TD and ASD groups in chronological age, *F*(1,41) = 5.86, *p* = .02. Children in the ASD group had been previously diagnosed with ASD through various sources (e.g., formal evaluations through an autism center or school diagnosis). Diagnosis was confirmed for the current study with module 3 of the ADOS-2 [49]. The ADOS-2 was administered by an individual trained to research reliability on administration, scoring, and interpretation of the measure. Participant information can be found in Table 1.

**Table 1:** Participant characteristics including: IQ (WASI), age, gender, SRS-2T-score, and ADOS-2 severity scores for the ASD group. Reprinted from [12]

Group	Participants	WASI (full scale)	Age	Gender	SRS-2 SCI *T* score	SRS-2 RBB *T* score	ADOS-2 Severity score
ASD	20	*M* = 107.35 SD = 16.27	*M* = 27.56^a^ SD = 7.20	19 M 1 F	*M =* 71.26^b^ SD *=* 12.25	*M* = 69.63^b^ SD = 11.40	*M* = 6.88 SD = 2.05
TD	23	*M* = 111.60 SD = 15.50	*M* = 21.68^a^ SD = 6.65	22 M 1 F	*M* = 48.52^b^ SD = 6.97	*M* = 50.69^b^ SD = 9.38	N/A

^a^*p* = .02

^b^*p* = < .0001

### Behavioral measures

Participants’ caregivers completed the Social Responsiveness Scales (SRS-2) [50], which measures social responsiveness and behavior. We also tested for overt motivational or affective differences between groups for each condition. To accomplish this, children (*N* = 21 TD, 19 ASD) completed a 1–7 Likert rating scale of how much they enjoyed the game (1 = “I do not like this game,” and 7 = “I love this game”) after each block. Participants also completed a 1–7 Likert scale about their perception of getting correct answers (1 = “I never got correct answers,” and 7 = “I always got correct answers”). In reality, the ratio of correct versus incorrect answers was predetermined and controlled by experimental design, and the rating was used to verify that the groups did not differ in their perception that they were obtaining correct answers.

### EEG recording

Participants wore a standard, fitted cap (Electrocap International) with 33 silver/silver-chloride (Ag/AgCl) electrodes placed according to the extended international 10–20 system. Continuous EEG was recorded with a NeuroScan 4.5 System with a reference electrode at Cz and re-referenced offline to the average activity at left and right mastoids. Electrode resistance was kept under 10 kΩ. Continuous EEG was amplified with a low pass filter (70 Hz), a directly coupled high-pass filter (DC), and a notch filter (60 Hz). The signal was digitized at a rate of 250 samples per second via an Analog-to-Digital converter. Eye movement artifacts and blinks were monitored via horizontal electrooculogram (EOG) placed at the outer canthi of each eye and vertical EOG placed above and below the left eye. Trials were time locked to the onset of the feedback stimulus. To measure reward anticipation, the baseline period was − 2200 to − 2000 ms, and the data were epoched from − 2200 to 100 ms. To measure reward processing, the baseline period was − 200 to 0 ms, and the data were epoched from − 200 to 800 ms. The interval between trials was varied between 1800 and 2000 ms. Trials with no behavioral response, or containing electrophysiological artifacts, were excluded from the averages.

Artifacts were removed via a four-step process. Data were visually inspected for drift exceeding +/− 200 mV in all electrodes, high frequency noise visible in all electrodes larger than 100 mV, and flatlined data. Following inspection, data were epoched and eyeblink artifacts were identified using independent component analysis (ICA). Individual components were inspected alongside epoched data, and blink components were removed. To remove additional artifacts, we utilized a moving window peak-to-peak procedure in ERPlab [51], with a 200-ms moving window, a 100-ms window step, and a 150-mV voltage threshold. Our final analyses for reward anticipation included 20 children with ASD and 23 TD children, and our final analyses for the reward processing included 19 children with ASD and 23 TD children.

Time-frequency decomposition was performed to compute event-related spectral perturbations (ERSP). ERSP measures changes in EEG power from the baseline period at a specific frequency (or frequency band) and time [52]. ERSPs were calculated using the “newtimef” plugin in EEGlab (version 12.0.2.6b) and MATLab (version R2014a). Standard settings within EEGlab newtimef were used (cycles set at 1.4, .5). This procedure yields a time × frequency transform with numbers for each time point, frequency, and trial. We utilized a linear space for frequency (1 Hz for reward processing and 2 Hz for reward anticipation). Alpha-band activity was operationalized as the average activity between 8 and 12 Hz, and theta-band activity was operationalized as the average activity between 4 and 6 Hz. Note that the theta-band was operationalized in this way (e.g., 4–6 Hz) to avoid overlap with the alpha band.

## Results

Data were analyzed using JMP (version 11.0). We used repeated measures analysis of variance (ANOVA) to test for differences between conditions and caudality (anterior-posterior scalp locations). Greenhouse-Geisser-corrected degrees of freedom are reported to account for violations of sphericity.

### Behavior

As reported in [12], no significant differences were found between groups on children’s Likert ratings of liking the game, *F*(1,39) = .72 ns, or perception of generating correct answers, *F*(1,39) = .95 ns. As expected, significant differences were found between groups on the SRS-2 social subscale, *F*(1,41) = 64.27 *p* < .001, and the repetitive behavior subscale, *F*(1,41) = 38.23 *p* < .001, with children with ASD scoring significantly higher on both subscales compared to TD children.

### ERSP

#### Reward anticipation

ERSP data during the feedback anticipation period were measured as the mean activation during the time period prior to feedback onset (e.g., − 2200 to 100 ms) with the time period from − 2200 to 2000 ms as the baseline. Alpha-band activity (8–12 Hz) was measured for the following electrodes: F3/F4, C3/C4, P3/P4, and T5/T6. For analysis concerning alpha asymmetry, log power in the left hemisphere was subtracted from the right hemisphere. Therefore, positive values indicate more right-hemisphere activity, whereas negative values indicate more left-hemisphere activity. Electrodes were chosen due to our and other groups’ previous research on event-related potential (ERP) measures of reward anticipation [12, 53].

##### Alpha band

**Fig. 2:**
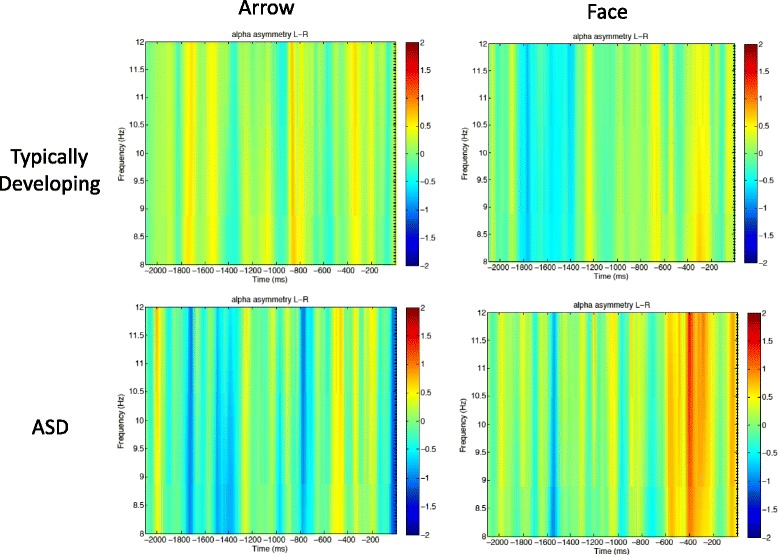
Anticipatory alpha asymmetry (left minus right) in temporal electrode locations. Note that negative values indicate more left dominant alpha supression, while positive values indicate more right dominant alpha supression. Data from typically developing children is shown in the top row, and data from children with autism is shown on the bottom row. Activity prior to nonsocial (arrow) stimuli is shown on the left, and activity prior to social (face) stimuli is shown on the right

A 2 (group) ×  2 (condition) ×  4 (electrode position) ANOVA was conducted. A marginal main effect of electrode, *F*(3,120) = 2.61, *p* = .059 was observed. No other interactions or effects were observed. Although the effect of electrode position was marginal, we conducted exploratory analysis of each electrode position (e.g., frontal, central, parietal, temporal). Therefore, these results should be interpreted cautiously. An interaction was observed between group × condition in the temporal electrodes, *F*(1,40) = 3.96, *p* = .05. Follow-up tests revealed a marginal effect of condition for children with ASD, *F*(1,40) = 3.78, *p* = .058, such that more left-hemisphere suppression was observed for the arrow (versus face) condition. Follow-up tests also revealed a marginal effect of the face condition, *F*(1,40) = 3.55, *p* = .06, such that TD children had greater left-hemisphere suppression in the face condition compared to children with ASD. No effects or interactions were observed in the other electrode positions. ERSPs for reward anticipation in the alpha band are shown in Fig. 2.

##### Brain and behavior correlations

**Fig. 3:**
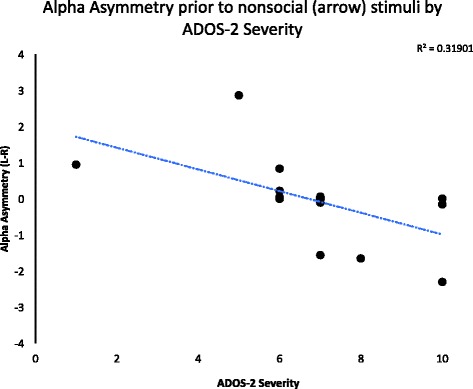
Correlation between anticipatory alpha asymmetry to nonsocial stimuli and ADOS-2 severity scores. Note that negative values for alpha asymmetry indicate more left dominent alpha supression, and higher ADOS-2 scores indicate more severe symptoms of autism

In order to explore whether EEG activity was related to behavioral and parent report measures of social responsiveness and severity of ASD symptoms, correlations were conducted. For the alpha band, correlations were conducted using EEG asymmetry (left to right) in the temporal electrodes (as the other positions did not reveal any significant effects or interactions) and continuous measures of autism symptoms. For individuals diagnosed with ASD, correlations were run using alpha asymmetry and severity score on the Autism Diagnostic Observation Schedule, second edition [49]. A significant correlation was observed between alpha asymmetry prior to nonsocial stimuli and severity score on the ADOS-2, *F*(1,16) = 7.49, *p* = .014, *R*^2^ = .27, such that individuals with more severe ASD evidenced greater left-dominant alpha band suppression and those with less severe ASD evidenced less left-dominant alpha band suppression prior to viewing nonsocial stimuli. Bonferroni-corrected threshold probability for statistical significance was .025 for these correlations. Correlations are shown in Fig. 3.

#### Feedback/reward processing

ERSP data during the feedback/reward processing period was measured as the mean activation during the time period immediately after feedback onset (i.e., − 200 to 800 ms) with the time period from − 200 to 0 ms as the baseline. Two separate bands of activation were measured: alpha-band activity (8–12 Hz) and theta-band activity (4–6 Hz). Mean activation was measured for the midline electrodes Fz, Cz, and Pz and was averaged across electrodes. These electrodes were chosen based on previous investigations on the FRN and theta band [38]. Trials were separated by whether participants received “correct” versus “incorrect” feedback (e.g., whether they were rewarded or not). However, it is important to note that our task was set up such that whether or not participants got a reward or not was pre-programmed and thus did not actually depend on participant response.

##### Alpha band

**Fig. 4:**
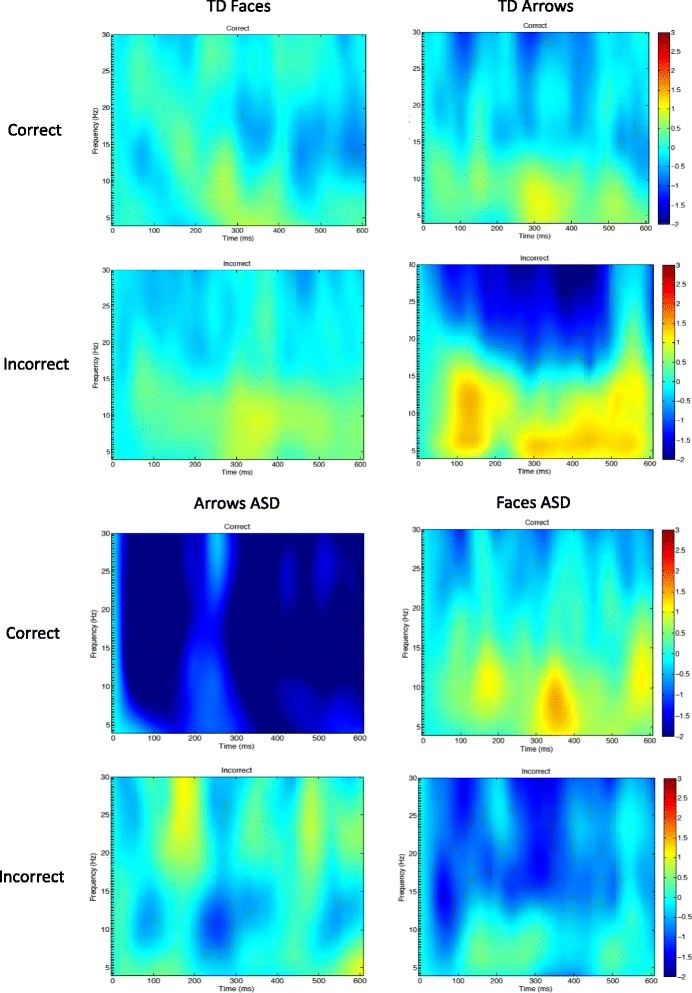
**a** Post-feedback ERSP for typically developing (TD) children in the theta (4–6 Hz) and alpha band (8–12 Hz). Activity after social (face) stimuli is shown on the left, and activity after nonsocial (arrow) stimuli is shown on the right. Activity after “correct” feedback is shown in the top row, and activity after “incorrect” feedback is shown in the bottom row. **b**. Post-feedback ERSP for children with ASD children in the theta (4–6 Hz) and alpha band (8–12 Hz). Activity after social (face) stimuli is shown on the left, and activity after nonsocial (arrow) stimuli is shown on the right. Activity after “correct” feedback is shown in the top row, and activity after “incorrect” feedback is shown in the bottom row

A 2 (group) ×  2 (feedback) ×  2 (condition) ANOVA was conducted. A main effect of group was observed, *F*(1,40) = 6.5, *p* = .01, such that children with ASD had more alpha suppression (8–12 Hz) during reward processing than TD children regardless of condition or feedback type. No other main effects or interactions were observed. ERSPs for post-feedback alpha are shown in Fig. 4.

##### Theta band

A 2 (group) ×  2 (feedback) ×  2 (condition) ANOVA was conducted. A main effect of group was observed, *F*(1,46.28) = 5.4, *p* = .02 such that TD children had more activity in the theta band (4–6 Hz) during reward processing than children diagnosed with ASD. No other main effects or interactions were observed. ERSPs for post-feedback theta are shown in Fig. 4.

##### Brain and behavior correlations

Correlations were conducted for reward processing for both the alpha and theta bands and measures of autism severity for the ASD group (ADOS-2).

##### Alpha band

For activity in the alpha band, a negative correlation was observed between alpha suppression in response to “correct” responses in the face condition and ADOS-2 severity score, *F*(1,16) = 5.64, *p* = .03, *R*^2^ = .21. Individuals with more severe ASD had less alpha suppression after “correct” feedback in the face condition, and those with less severe ASD had more alpha suppression after “correct” feedback in the face condition. However, as four conditions (correct/incorrect for both social and nonsocial conditions) were analyzed, and this correlation did not reach significance under the Bonferroni-corrected threshold for statistical significance level of .0125.

##### Theta band

**Fig. 5:**
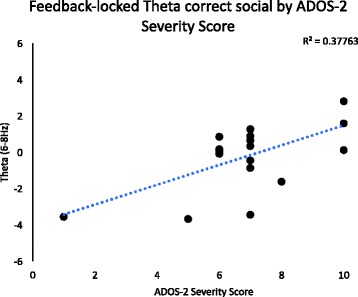
Theta (4–6 Hz) correlation between post-stimulus theta after “correct” social (face) feedback and ADOS-2 severity score. Note that higher ADOS-2 scores indicate more severe ASD

For activity in the theta band, a significant positive correlation was observed between ADOS-2 severity score and theta-band activity for “correct” feedback in the face condition, *F*(1,16) = 9.7, *p* = .006, *R*^2^ = .33. Individuals with more severe ASD evidenced more theta activity after “correct” feedback in the face condition, and those with less severe ASD evidenced less theta activity. Correlations for post-feedback theta are shown in Fig. 5.

#### Anticipatory and feedback correlations

To better understand whether individuals with ASD in the current study might be experiencing both reduced reward anticipation and sensory/attentional hypoactivation during reward processing, correlations between pre-stimulus alpha and post-stimulus alpha were conducted. Note that the threshold for significance was set at .00625 (.05/8), as correlations were run between pre-stimulus alpha in two conditions and post-stimulus alpha in four conditions—leading to eight independent correlations. A significant negative correlation was observed between pre-stimulus alpha in the face condition and post-stimulus alpha in the correct arrows condition, (*F* = 16.85, *p* = .0007, *R*^2^ = .45). That is, children with ASD with *greater* left-hemisphere alpha suppression when anticipating faces had *less* alpha suppression in response to correct arrows after feedback. No other significant correlations were observed.

## Discussion

Results of the current investigation have implications for neural mechanisms in ASD and increase our understanding of how different theoretical perspectives may be simultaneously accurate. We analyzed event-related spectral perturbations (ERSPs) during a reward task designed to explore both reward anticipation and processing for social and nonsocial rewards. We focused on activity in the alpha (8–12 Hz) and theta (4–6 Hz) bands, due to their hypothesized role in reward anticipation, reward processing, and general cognitive engagement.

### Reward anticipation

We analyzed alpha asymmetry during reward anticipation, as alpha asymmetry has been thought to reflect approach motivation and anticipation of upcoming rewards [23–25]. Our findings largely agree with previous literature, although the topographic distribution of our findings differs from previous reports. Previous literature has related increased approach motivation with alpha asymmetry in frontal regions (e.g., [23, 54]), whereas the current investigation found significant results only in temporal electrode locations. These differences may be largely attributable to the stimuli used in the current study. Previous work investigating alpha asymmetry has utilized reward paradigms with monetary incentives (e.g., [23, 54]), whereas the current paradigm used social (face) versus nonsocial (scrambled face) reward information. Given that face stimuli are thought to be processed in temporal locations [55], our topographic findings are consistent with literature on face processing.

Though exploratory, our findings provide evidence in favor of the social motivation hypothesis of ASD during reward anticipation. That is, children with ASD had marginally less left-dominant alpha activity when anticipating faces compared to their TD peers. Further, children with ASD-evidenced greater left-dominant alpha activity when anticipating arrows versus faces. Interestingly, these findings not only suggest that children with ASD have less anticipatory activity (compared to their TD peers) prior to viewing faces but also suggests that children with ASD have greater approach motivation prior to viewing non-face versus face stimuli.

It may be the case that, compared to their TD peers, children with ASD have over-active reward anticipation prior to nonface stimuli. It is possible that reward value is placed on nonsocial stimuli at the expense of social information. This view is further corroborated by correlations between ASD severity and alpha asymmetry. Children with ASD who scored higher on the ADOS-2 severity score algorithm (indicating more severe ASD) had more left-dominant alpha suppression prior to viewing nonsocial stimuli (arrows), and those who had less left-dominant alpha suppression prior to nonsocial stimuli had lower ADOS-2 severity scores. Taken together, these findings are in line with what would be expected given the social motivation hypothesis, but extend the hypothesis with evidence that perhaps social deficits in ASD are due to both *hypo*active reward anticipation for social information and *hyper*active anticipation for nonsocial stimuli.

### Reward processing

#### Theta band

We analyzed reward processing by looking at activity in the theta band (4–6 Hz) after feedback was provided. We separated trials based on whether participants got “correct” versus “incorrect” feedback. It is important to note that we did not observe significant effects of feedback type (e.g., “correct” versus “incorrect”), which differs from previous investigations of reward processing in ASD [40, 41]. However, one explanation for this may be that in the current paradigm, “incorrect” feedback did not lead to participants losing rewards but rather meant that participants did not get a reward on that trial. So, rather than having “win” versus “loss” conditions, it is more accurate to conceptualize our paradigm as containing “win” versus “no-win” conditions. Further, previous studies have utilized monetary rewards [40, 41] rather than contrasting social versus nonsocial reward. Taken together, it seems likely that these differences in paradigm design may contribute to why we did not observe significant effects of feedback type in the current study.

Although we hypothesized a group by condition interaction, we observed a main effect of group such that TD children evidenced more theta activity compared to children with ASD regardless of whether rewards contained social or nonsocial information. This suggests that during reward processing, TD children have more reward-related oscillatory brain activity than children with ASD. This is particularly interesting given that we observed a different pattern during reward anticipation (e.g., children with ASD evidenced less reward-related activity when anticipating social versus nonsocial reward feedback). However, we believe this may illuminate an important aspect of neural functioning in ASD. It is possible that children with ASD experience less anticipation for social rewards than TD children, but less overall reward-related activity once rewards are presented.

Interestingly, we observed a significant positive correlation between theta activity and severity of ASD (via ADOS-2 severity scores) in the group with ASD. Specifically, we found that children who evidenced less theta activity after correct feedback in the social condition had less severe symptoms of ASD than those who had more theta activity in response to correct feedback in the social condition. This suggests that children who are more responsive to positive social feedback have more severe ASD symptoms compared to those who are less responsive to this feedback, which may provide preliminary evidence in favor of the IWH.

Thus, although we did not observe differences in reward processing for social versus nonsocial conditions between groups, correlations involving individual scores may provide a more detailed picture of what is occurring on an individual subject level. Within the ASD group, participants who were more responsive to correct social feedback had more ASD symptomology. Given that previous investigations of theta-band activity after rewards have found greater activity in response to “incorrect” versus “correct” feedback, it is possible that these results point to a dysfunction in how children with ASD process rewards. It is possible that children with ASD who experience greater theta activity in response to “correct” social feedback are experiencing hyperreactivity or are overwhelmed by the “correct” social feedback. We hypothesize that perhaps children with more severe ASD symptoms may be overwhelmed by social and/or emotional feedback (e.g., the feedback involved a smiling face and was thus both social and emotional). However, the current study was not designed to assess children’s subjective experiences of the reward feedback, but future studies may consider adding a child interview in order to help shed light on these types of findings.

#### Alpha band

We also analyzed activity in the alpha (8–12 Hz) band after feedback. However, we note that activity in the alpha band is not typically thought to reflect reward processing but rather to reflect general cognitive engagement. Therefore, we hypothesized that if children with ASD tend to experience stimuli as overly intense, we might observe greater suppression in the alpha band for children with ASD during feedback. As expected, we found that children with ASD evidence greater suppression in the alpha band after feedback compared to TD children regardless of condition. This is particularly interesting given that results in the theta band held the opposite pattern (e.g., greater activity for TD children compared to those with ASD). Taken together, these findings provide novel evidence that simultaneously supports both the social motivation hypothesis and the overly intense world hypothesis.

#### Anticipation and processing

To explore the potential relationship between reward anticipation and over-responsivity in ASD, correlations between pre-stimulus alpha asymmetry and post-stimulus alpha were conducted. We found that children with ASD who evidenced *greater* left-hemisphere alpha suppression when anticipating faces had *less* alpha suppression in response to correct arrows after feedback. This provides preliminary evidence for an important relationship between reward anticipation and hyperresponsivity in ASD. That is, children with ASD who evidenced greater approach motivation prior to face stimuli had less evidence of hyperresponsivity after correct feedback for nonsocial stimuli, and conversely, children with ASD with less approach motivation prior to face stimuli had more evidence of hyperresponsivity after correct feedback for nonsocial stimuli. These findings provide preliminary evidence that individuals with ASD may experience both the SMH and IWH.

## Limitations

There are limitations to the current study, which must be taken into account when interpreting the results. Children with ASD who took part in the current study all had cognitive abilities within the average range and can therefore be considered relatively high functioning. Therefore, our results may not be generalizable to other children with ASD who experience greater impairment. Further, although we hypothesize that activity in the alpha band after reward feedback reflects cognitive engagement, and a global increase in alpha band activation may reflect over-reactivity of attentional systems in ASD, the current study did not ask children about their subjective experiences. Therefore, results concerning the alpha band after reward feedback may be considered exploratory rather than confirmatory and should be replicated in future studies. Finally, although we utilized ERSP in order to measure changes in spectral power that are both phase-locked and nonphase-locked, the current study cannot rule out contributions of large-amplitude brain activity (e.g., P300) to our post-stimulus alpha and theta results. That is, we cannot claim that post-stimulus changes in alpha or theta-band spectral power are not due, in part, to attentional processes related to the P300. However, even if our post-stimulus findings are related to attentional processes, it does not negate the importance of our results for understanding neural processes in children with ASD.

## Conclusions

To our knowledge, this is the first study to measure ERSP activity in children with and without ASD in response to social versus nonsocial rewards. Our results provide further evidence that children with ASD have anticipatory reward deficits for social information and may anticipate nonsocial rewards more than social rewards. This is an intriguing possibility and has implications for neural mechanisms of ASD and potential targets for early intervention. That is, if social motivation deficits in ASD can be traced back to over-active anticipation of nonsocial rewards, it will be particularly important to deliver early interventions designed to increase the reward value of social information. Increasing the reward value of social information and social interactions can be done using a variety of methods, including taking the perspective of the child with ASD (e.g., [56]) and setting up the environment to reward social initiation (e.g., [57]).

Along with considerations related to intervention for children with ASD, it is important to investigate how it has come to be that children with ASD appear to be less rewarded by social information. Although the current study represents a developmental snapshot and thus cannot empirically address this question, it is important to consider the origins of the brain responses we report here to promote understanding of social perception development in ASD.

As detailed by de Haan, Humphreys, and Johnson in the “interactive specialization” hypothesis [58], typically developing infants’ preference for faces may be driven by subcortical biases which, in turn, cause infants to frequently look at faces. As a product of looking at faces so frequently, cortical systems develop to be “specialized” for faces. Retrospective studies of infants who do versus do not go on to develop ASD found that looking time to faces does not differ between groups at 2 months of age, but does differ at 6 months of age such that infants who go on to develop ASD initially look at faces less than their TD peers [59]. Connecting these findings to our own, we hypothesize that although the initial subcortical systems which cause infants to be biased towards faces exist in infants with ASD, neural pathways that are involved in the latter stages of “interactive specialization” are not functioning appropriately, which causes individuals with ASD to not connect face stimuli with the reward system or preferential attention during development.

We hypothesize that is attributable to either (a) infants with ASD not connecting positive emotions to faces, which for TD children turns the initial subcortical bias into something more cortically based for TD children, and causes TD children (and adults) to be rewarded by social stimuli, or (b) initial cortical bias to attend towards faces is overwhelming or aversive for infants with ASD, causing them to begin avoiding face stimuli, which in turn causes a developmental cascade of missed social opportunities and thus lack of neural specialization and reward anticipation.

It is important for future investigations to shed light on the developmental processes in ASD that lead social information to be less rewarding, as understanding when and how developmental processes deviate from TD children will assist in developing both behavioral and medical interventions. Brain measures in response to faces in young infants and children who are at risk for ASD versus those not at risk may provide experimental data that inform theory.

The design of the current study allowed us to investigate neural activation both during reward anticipation and reward processing. Reward processing results in the theta band indicate a global *hypo*activation for children with ASD regardless of whether rewards are social versus nonsocial. Results of activity in the alpha band during reward processing provide preliminary evidence that children with ASD may experience *hyper*activation of cognitive engagement during reward processing. It is possible that the attentional or sensory processing systems are over-active in ASD during reward processing at the expense of more typical reward processing systems. However, the current investigation was not designed to directly parse the relative contribution of attention, reward, and sensory processing. Future investigations may consider combining temporally sensitive techniques with spatially sensitive measures (e.g., combined EEG and fMRI) in order to maximize our understanding of both temporal activation and responses from discrete brain areas.
